# Visible and Extended Near-Infrared Multispectral Imaging for Skin Cancer Diagnosis

**DOI:** 10.3390/s18051441

**Published:** 2018-05-05

**Authors:** Laura Rey-Barroso, Francisco J. Burgos-Fernández, Xana Delpueyo, Miguel Ares, Santiago Royo, Josep Malvehy, Susana Puig, Meritxell Vilaseca

**Affiliations:** 1 Centre for Sensors, Instruments and Systems Development, Technical University of Catalonia, Terrassa 08222, Spain; francisco.javier.burgos@upc.edu (F.J.B.-F.); xana.delpueyo@upc.edu (X.D.); miguel.ares@oo.upc.edu (M.A.); santiago.royo@upc.edu (S.R.); meritxell.vilaseca@upc.edu (M.V.); 2 Dermatology Department of the Hospital Clinic of Barcelona, IDIBAPS; Barcelona 08036, Spain; jmalvehy@gmail.com (J.M.); susipuig@gmail.com (S.P.)

**Keywords:** InGaAs camera, multispectral imaging, infrared, skin cancer, melanoma

## Abstract

With the goal of diagnosing skin cancer in an early and noninvasive way, an extended near infrared multispectral imaging system based on an InGaAs sensor with sensitivity from 995 nm to 1613 nm was built to evaluate deeper skin layers thanks to the higher penetration of photons at these wavelengths. The outcomes of this device were combined with those of a previously developed multispectral system that works in the visible and near infrared range (414 nm–995 nm). Both provide spectral and spatial information from skin lesions. A classification method to discriminate between melanomas and nevi was developed based on the analysis of first-order statistics descriptors, principal component analysis, and support vector machine tools. The system provided a sensitivity of 78.6% and a specificity of 84.6%, the latter one being improved with respect to that offered by silicon sensors.

## 1. Introduction

Due to the uncontrolled growth of abnormal cells in skin cancer, chromophores such as melanin, hemoglobin, and water might differ among tumors of different etiologies; thus, skin cancer lesions can be identified clinically when a lesion changes its color, increases its size or thickness, and gets an unusual texture or its outline becomes irregular [1,2]. In this context, multispectral imaging systems, which provide precise quantification of spectral, colorimetric, and spatial features, have been employed over the last few years to analyze spectral and colorimetric properties of the skin reliably and non-invasively [3,4]. Specifically, they have also been used to improve the detection of skin cancer, especially melanoma, which is the most aggressive and lethal form.

In 2005, Tomatis et al. [5] developed an automated multispectral imaging system for the diagnosis of pigmented lesions. The device consisted of a spectrophotometer with a light source, a monochromator, and a bundle of optical fibers coupled to a probe head. The spectral range from 483 nm to 950 nm was covered with 15 spectral bands. A region-growing algorithm segmented the lesions and extracted descriptors to be used as input for setting and testing a neural network classifier.

Some years later, Kuzmina et al. [6] used a multispectral system that incorporated halogen lamps and filters from 450 nm to 950 nm with a spectral bandwidth of 15 nm. They observed that wavelengths closer to the infrared (IR) range penetrate deeper into the skin, resulting in decreased contrast due to higher light scattering. However, in the case of melanomas the contrast at 950 nm was found to be much higher, indicating considerably deeper structural damage.

In 2012, Bekina et al. [7] analyzed lesions under a multispectral system with four different spectral bands, each one to obtain information from specific structures of the skin: 450 nm for superficial layers, 545 nm for blood distribution, 660 nm for melanin detection, and 940 nm for the evaluation of deeper skin layers. Then, a ratio was calculated between the intensities of green light (545 nm), where the hemoglobin absorption is high, and red light (660 nm), where it is low. It was proven that pathological tissues showed higher values of this index than the surrounding skin as a consequence of having higher blood content. The same authors developed a similar system [8] that consisted of a multispectral imaging system with a CCD imaging sensor and a liquid crystal tunable filter (LCTF) (with spectral bands from 450 nm to 950 nm in steps of 10 nm) (Nuance EX), a spectral optimized lens, and internal optics. The illumination system was a ring of halogen lamps with a polarizer orthogonal to the camera in order to remove the artifacts caused by light reflection. In order to differentiate between melanoma and nevi, a new parameter was suggested based on skin optical density differences at three wavelengths: 540 nm, 650 nm, and 950 nm.

Additionally, Jakovels et al. [9] used principal component analysis (PCA) of multispectral imaging data in the wavelength range from 450 nm to 950 nm for distant skin melanoma recognition, which resulted in clear separation between malignant melanomas and pigmented nevi.

Delpueyo et al. [10] also proposed a light-emitting diodes (LEDs)-based multispectral imaging system with eight different wavelengths (414–995 nm) but using the analysis of the spatial distribution of color and spectral features through descriptors based on the first-order statistics of the histogram to improve the detection of skin cancer lesions, specifically melanomas and basal cell carcinomas.

Recently, Kim et al. [11] have investigated the potential of mobile smartphone-based multispectral imaging for the quantitative diagnosis and management of skin lesions. The authors miniaturized a spectral imaging system so that it could be attached to a smartphone, allowing users to obtain ten images sequentially within a range of wavelengths from 440 nm to 690 nm, with one white-light image. The results suggested that smartphone-based multispectral imaging and analysis had great potential as a healthcare tool for quantitative mobile skin diagnosis.

Despite the fact that sensitivity and specificity obtained with the former multispectral imaging systems based on silicon imaging sensors have reached similar values to those obtained by experienced dermatologists through dermoscopy [10,12], they have not yet superseded histological examination. In fact, this continues to be the clinical gold standard, providing diagnostic confirmation after surgical excision of the tumor.

Digital cameras based on silicon have a spectral response in the visible (VIS) up to the near-infrared (NIR) (900 nm–1000 nm). However, multispectral, extended, near-infrared (exNIR) optical imaging is nowadays available thanks to new indium gallium arsenide (InGaAs) cameras with high quantum efficiency within 900 nm–1600 nm. This has sparked interest among biologists in this relatively unexplored spectral region also known as “the second near-infrared window” (900 nm–1400 nm) [13].

Going further than 900 nm into the exNIR enables deeper in vivo optical imaging as photons at these wavelengths penetrate deeper into living tissue [14]; this could be a hint to explore further this spectral region as a means of improving skin cancer diagnosis and prognosis, as it may release information about how tissues are damaged due to ultraviolet radiation, water content, and other factors that might be different in benign and malignant lesions. For instance, due to the increased absorption of water in this spectral range, spectral images in the exNIR can provide information about the presence of angiogenesis, a tumorous growth of blood vessels around the malignant lesion.

In fact, the near-infrared region has been widely utilized in the past decade, and a number of clinical imaging applications have already been developed [15].

In this study, we investigate the possibilities of a multispectral imaging system based on an InGaAs camera and light-emitting diodes (LEDs) for the detection of skin cancer, especially melanomas, in the exNIR range, specifically from 995 nm to 1613 nm. Although InGaAs sensors tend to be noisier than CMOS or CCD sensors and the deeper scattering at exNIR wavelengths can reduce image contrast, we consider that the study of this unexplored spectral range can bring very interesting results to the scientific community.

## 2. Experimental Setup and Clinical Measurements

**Figure 1:**
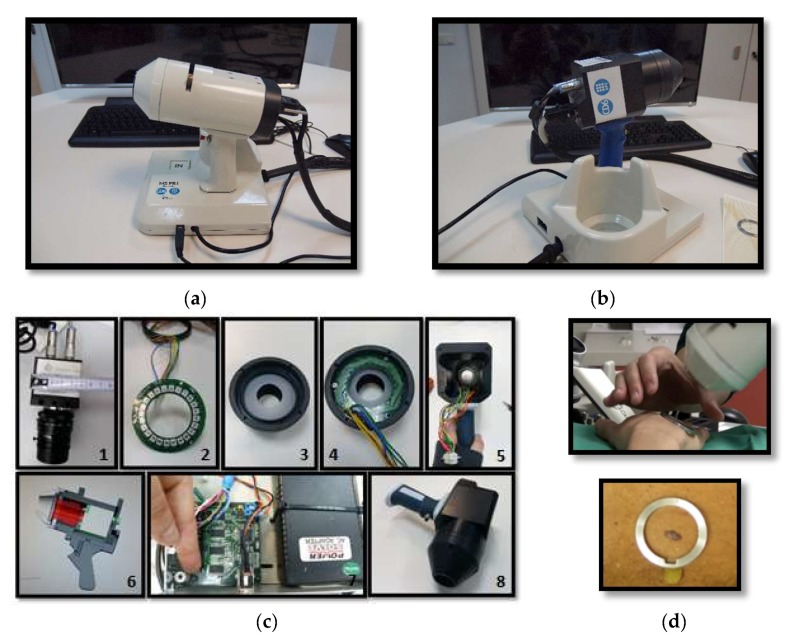
(**a**) General view of the previously developed handheld VIS-NIR multispectral device. (**b**) General view of the handheld exNIR multispectral device. (**c**) Components of the exNIR imaging system: 1—InGaAs camera, 2—Ring of LEDs, 3—Tip of PVC cone, 4—Ring of LEDs placed at the tip. 5—Handheld case, 6—AutoCad design, 7—Electronic control, and 8—Handheld case with camera inside. (**d**) Clinical measurement and metallic ring, which is glued to the patient’s skin for the tip of both systems to place them in the same position and parallel to the skin, without making any contact.

**Figure 2:**
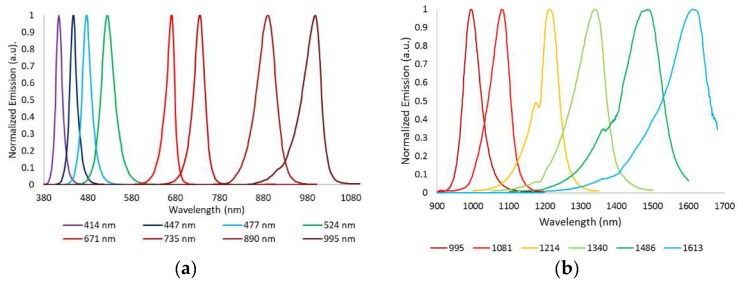
Normalized spectral emission of the LEDs in (**a**) the VIS-NIR and (**b**) exNIR ranges.

The device developed to perform exNIR multispectral imaging of skin cancer is depicted in Figure 1b,c. It integrates a 16-bit depth InGaAs camera (Hamamatsu C10633-23, Hamamatsu Photonics, Shizuoka, Japan) with spectral sensitivity from 900 nm to 1600 nm, readout speed of 50 fps, and 320 × 256 pixels together with a Kowa LM12HC-SW 1.4/12.5 mm short-wave infrared (SWIR) lens with high transmission from 800 nm to 2000 nm (Kowa Company, Ltd., Aichi, Japan). Additionally, a LED-based light source was built on a cylinder of polyvinyl chloride (PVC) including high power Surface Mounted Displays (SMD) LEDs with peak wavelengths at 995 nm, 1081 nm, 1214 nm, 1340 nm, 1486 nm, and 1613 nm (Figure 2b); they were selected in accordance with the absorption curves of the principal chromophores of the skin, such as bilirubin, hemoglobin, and water, especially taking into account their most representative minimums and maximums, and the spectral bands with considerable differences among them allowing characterization of the tissue constituents [10]. The tip of the cylinder is a cone with an opening of 20 mm × 20 mm and a diffuser. Four LEDs per spectral band were included in the light source with a separation of 90° among them to ensure a uniform illumination over the skin; a total amount of 24 LEDs was finally placed on the ring. All parts were assembled in a handheld configuration with a trigger to start the acquisition. A base was also designed and constructed to hold the multispectral system when it was not being used. It incorporated a calibrated reference at the bottom that consisted of the Neutral 5 gray color of an X-Rite ColorChecker^®^ (Grand Rapids, MI, USA) with level of reflectance similar to that of the skin in the exNIR range. The degradation of the reference over time was controlled by visual inspection and spectrally by means of a spectrometer. If the reference showed dirty areas or scratches or its reflectance spectrum presented variations up to 5% with respect to the spectrum when it was brand new, it was replaced.

**Figure 3:**
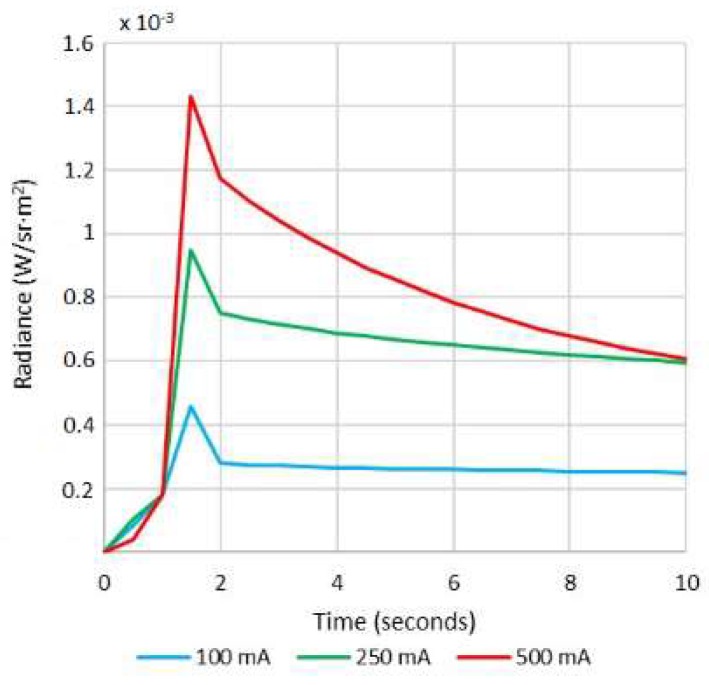
Radiance (W/sr·m^2^) of the 414 nm LEDs at different forward currents measured every second over 10 s.

The exNIR system was used together with another multispectral imaging system previously built [10] for capturing reflectance and color features in the VIS and NIR ranges (Figure 1a). In this case, the camera integrated in the multispectral head is a 12-bit depth DMK 23U445 with a 1/3″ CCD sensor of Sony ICX445ALA with 1280 × 960 pixels of resolution and readout speed of 30 fps; it represented an advantage over the spatial resolution of the exNIR camera, which limited the performance of the analysis in this spectral range. The lens coupled was a Schneider-Kreuznack Cinegon with spectral sensitivity from 400 nm to 1000 nm and a working distance from infinite to 20 mm, which allowed focusing the skin lesions at a distance of 40 mm with a field of view of 15 mm × 20 mm. As in the previous case, sequential multiplexed illumination is used by means of a ring of 32 LEDs (four per wavelength) with the following peak wavelengths: 414 nm, 447 nm, 477 nm, 524 nm, 671 nm, 735 nm, 890 nm, and 995 nm (Figure 2a). For each wavelength, the forward current of the LEDs was tuned to obtain a constant temporal behavior in terms of radiance. The final currents used for each wavelength were selected, taking into account a compromise between emission stability and the exposure time needed to make use of the whole dynamic range of the cameras: from 0 to 4095 digital levels in the case of the VIS-NIR system and from 0 to 65,533 in the case of the exNIR system. In general, measurements of radiance over time showed that LEDs needed at least two seconds to stabilize, and therefore this delay was added when any LED was switched on (Figure 3). Infrared LEDs used in the exNIR device showed a much more stable behavior than those in the VIS-NIR range; for this reason, the two-second delay was not implemented in this system. Despite that, the spectral assessment in the exNIR was less accurate than in the VIS-NIR, because exNIR LEDs presented a wider Full Width at Half Maximum (FWHM). In consequence, the information captured at each spectral band and then associated with the wavelength peak of each LED includes a broader range of data coming from more wavelengths.

A user-friendly acquisition software was built based on Borland Builder C++ to be used for physicians in a clinical environment. The software controls individually and synchronously the emission of the LEDs and the acquisition of spectral images. The software interface asks for a daily calibration to guarantee accurate measurements at all wavelengths along LEDs lifecycle. It involves the acquisition of images of the neutral reference (Neutral 5) at each spectral band and at ten different exposure times to adapt the dynamic range of the system to every skin phenotype, thus avoiding saturated or noisy areas. This set of calibration images is used later for calculating the reflectance images from the raw spectral images (see next section). Then, a measurement over the actual skin lesion can be taken, and the system will make a sequence of acquisitions at every spectral band. In the case of the multispectral system with LEDs in the VIS and NIR ranges, eight images are obtained, while for the one in the exNIR, six images of a lesion are taken. Each one is acquired with a given exposure time, chosen from a learning table among the ten exposure times previously used to take images of the reference. The exposure times are chosen by an algorithm with a target averaged digital level (DL) for each image that is about half of the dynamic range of the camera; in this way, saturated images are avoided. This active exposure time selection also contributes to compensating variations of LEDs’ power output due to their lifecycle. Another advantage of this algorithm is that it allows the evaluation of all kinds of skin, from brighter to darker ones. A set of dark current images also needs to be acquired by just making another calibration in a room in dark conditions, to take into account the straylight caused by internal reflections and noise sensed by the camera at the time of calculating the reflectance curve of a pixel area; the same ten exposure times previously used are applied. The straylight was measured sequentially switching on the LEDs, as during the calibration with the reference, in order to remove from the image the reflections caused by the inner surface of the PVC painted cone. Environmental illumination barely affects the measurement, because the tip of the devices is fully covered by the metallic ring and the patient’s skin. One lesion is fully analyzed when sequentially measured with the VIS-NIR and the exNIR multispectral imaging systems. Apart from the additional spectral information from 414 nm to 995 nm, the use of the VIS-NIR system allows to locate with higher accuracy some lesions that present blurry boundaries in the IR.

As a pilot study, the VIS-NIR and exNIR multispectral systems were used to analyze 39 nevi and 14 melanomas from Caucasian patients at the Hospital Clínic *i* Provincial de Barcelona (Barcelona, Spain). Patients could be seated or lying down on the hospital bed while capturing.

All of them provided written informed consent before any examination and ethical committee approval (Spanish Agency of Drugs and Clinical Products, document number 7576) was obtained. The study complied with the tenets of the 1975 Declaration of Helsinki (Tokyo revision, 2004). The lesions were diagnosed by dermatologists (SP and JM) using a commercial dermoscope and the confocal laser scanning microscope VivaScope^®^ 1500 from MAVIG GmbH (Munich, Germany). When malignancy was suspected, the lesion was excised and a histological analysis was carried out.

## 3. Data Processing

The images from the multispectral systems were processed through a graphical user interface (GUI) built in Matlab R2015a (The MathWorks Inc., Natick, MA, USA). It has different functions to make internal calibration algorithms and also to compute reflectance spectra that can be used to compare subtle differences between benign and malignant lesions. In order to calculate the reflectance images, the corresponding calibration images from the reference and dark current images are selected by the program. Then, for a given exposure time and wavelength, being different the exposure time for each wavelength, the reflectance at each pixel (*i*, *j*) is calculated as follows:

(1) $$R_{Lesion}\left(i,j\right)=k\frac{I\left(i,j\right)-\;I_{D}\left(i,j\right)}{I_{N}\left(i,j\right)-\;I_{D}\left(i,j\right)},$$

where $R_{Lesion}\left(i,j\right)$ is the spectral reflectance image; I(i,j), $I_{N}\left(i,j\right)$, and $I_{D}\left(i,j\right)$ contain the DLs of the raw, neutral gray reference and dark current images, respectively; and *k* is the calibrated reflectance of the neutral gray reference, given by the manufacturer.

Since the purpose was to obtain information about the lesions themselves and not from the whole reflectance images, which also contain spectral information about the surrounding healthy skin, every lesion was segmented. The segmentation of lesions from the IR images was a challenge, because in many cases they were hardly distinguishable from the surrounding skin because of the decreased contrast due to higher scattering at these wavelengths. In order to overcome this, two different algorithms of segmentation were used for each multispectral system.

**Figure 4:**
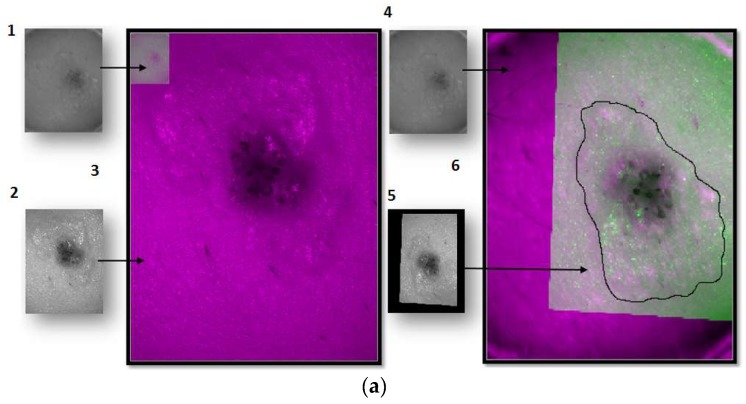

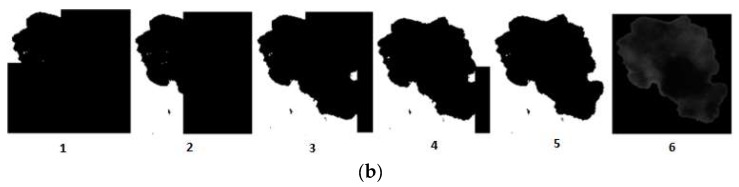
Steps of the segmentation algorithm for both multispectral imaging systems. (**a**) Example of how the segmentation is done for a lesion taken with the exNIR system: (1) Reflectance image taken with the exNIR device. (2) Reflectance image taken with the VIS-NIR camera. (3) Reflectance images from both cameras superimposed. Here, it can be seen how both systems take images at different resolutions, rotated with respect to each other a certain angle. (4) Reflectance image of the exNIR imaging system. (5) Reflectance image from the VIS-NIR system, to which a mathematical operation for correlation has been applied. (6) These last images were superimposed. (**b**) Example of how the segmentation is done for a lesion taken with the VIS-NIR imaging system: images correspond to the implementation of the Otsu method for each subimage. A mask for each of them is calculated in (1–4), and they are put together to form the (5) final mask for segmentation. (6) is the result of segmenting the lesion.

For the images of the lesion in the exNIR range, manual segmentation coupled with previous image registration was used. In this case, the corresponding images of the lesion taken in the VIS range were correlated to those captured by the exNIR multispectral system in order to overcome the constraint of the second device. This could be easily done by means of a mathematical transformation of the images matrices, since skin lesions were measured by both systems at the same position and in the same orientation thanks to a metallic ring were the tip of both systems could be attached to the patient’s skin (Figure 1d). The metallic ring was glued to the patient’s skin by means of a medical adhesive ring that was different for every patient; this combination prevents the systems from touching the skin. Afterwards, when the corresponding images were shown together, one on top of the other, manual segmentation was performed over the images in the exNIR range (Figure 4a).

The algorithm used for the images in the VIS-NIR range consisted of searching the DL threshold to establish those pixels belonging to the patient’s lesion (foreground) and those to the surrounding healthy skin (background). The DL threshold was calculated with the Otsu’s method, which maximizes the between-class variance of the lesion and the skin pixel values based on the intensity of the histogram [16]. A binary image is created according to the threshold, and pixels below are transformed into zeros and above into ones. This binary image can be used as a mask to be applied to all the spectral images and obtain the DLs corresponding to the pixels of the lesion. However, Otsu’s method is based on a global threshold that can only be used in lesions that are clearly different from the skin. In order to solve skin inhomogeneity, reflectance images were divided into four subimages, allowing different thresholds adapted to the local characteristics to be calculated (Figure 4b). The image used for performing VIS-NIR segmentation was taken at 414 nm, since this spectral band enhanced the detection of melanin, and therefore it extended the contour of the lesion to its real size.

Different parameters to discriminate between benign and malignant lesions were also calculated based on spectral features additionally to the reflectance images (Equation (1)). To avoid the influence of the patient’s healthy skin, another set of reflectance images were calculated by subtraction of the mean reflectance value of the patient’s skin from the reflectance images of the segmented lesion (Equation (2)). This was proposed as an empirical approach to improve the results, if possible, due to the different penetration depths of wavelengths owing to the different absorption of structures inside the tissue; however, in multispectral technology, images contain a mixture of light coming from not only a specific depth inside the tissue but also from the preceding layers due to backscattering.

Another two set of images were also computed by taking the logarithm of the latter ones, in order to obtain information in terms of absorbance, too.

(2) $$R_{Lesion-Skin}\left(i,j\right)=k\frac{I_{lesion}\left(i,j\right)-\;I_{D}\left(i,j\right)}{I_{N}\left(i,j\right)-\;I_{D}\left(i,j\right)}-mean\left(k\frac{I_{skin}\left(i,j\right)-\;I_{D}\left(i,j\right)}{I_{N}\left(i,j\right)-\;I_{D}\left(i,j\right)}\right),$$

At a second stage, a statistical analysis over the former images was carried out as a first approach to characterize the spatial features based on the histogram of the lesions and obtain further information for the classification algorithm. This analysis was envisaged to provide information about the distribution of digital levels at each spectral band and all over the sample rather than only taking into account the traditional statistical descriptors: mean (*µ*), maximum (*max*), minimum (*min*), and standard deviation (*σ*). Accordingly, first order statistics descriptors such as the energy (*En*), the entropy (*Ep*), and the third central moment (*µ*_3_) were obtained from the histogram of the segmented lesion in terms of the spectral reflectance and absorbance; these parameters have been shown to provide useful information about human features such as the iris pattern [17]. They are defined as follows:

(3) $$E_{n}=\overset{N-1}{\underset{i=\;0\;}{\sum }}P{\left(i\right)}^{2},$$

(4) $$E_{p}=-\overset{N-1}{\underset{i=\;0\;}{\sum }}P{\left(i\right)}^{2}\log\left[P{\left(i\right)}^{2}\right],$$

(5) $$\mu_{3}=\overset{N-1}{\underset{i=\;0\;}{\sum }}{\left(i-\mu \right)}^{3}P{\left(i\right)}^{2},$$

where P(i) is the value (frequency) of the intensity element i (bin) of the histogram and N is the number of levels that the histogram is divided into.

Energy is a numerical descriptor of uniformity that ranges between 0 and 1, reaching the maximum value for a constant image [18]. In regards to entropy, it is a well-known statistical measure of randomness, uncertainty, or disorder in image values, with 0 being the minimum value for a constant image and $\log^{2}\left(N\right)$ the maximum [18]. The third central moment or skewness, $\mu_{3}$, refers to the skewness of the histogram about its mean; it has a range of values between −1 and 1, positive for histograms skewed to the right, negative for the ones skewed to the left, and 0 for symmetric ones [18]. According to all this, these descriptors can be used to account for reflectance distribution features of skin lesions besides the more classical averaged spectral information obtained from traditional multispectral imaging systems.

## 4. Classification Algorithm

In order to determine which of the former statistical descriptors related with reflectance and absorbance values were useful to discriminate between malignant and benign tumors, as well as reflectance/absorbance minus the average of the healthy skin, scatter plots with the values for every lesion analyzed were evaluated. In total, there were 392 scatter plots, 224 from the VIS-NIR system and 168 from the exNIR. A Matlab-based classification algorithm was then used to find the best ones. The algorithm included the definition of upper and lower thresholds on the scatterplots that were experimentally set to delimit the area where benign lesions were prone to lay down. The lesions that fell outside the thresholds for at least one parameter were considered to be malignant (i.e., melanomas).

The classification algorithm worked as follows: after setting the thresholds of all parameters, they were ordered according to the number of malignant lesions that they allowed to classify. Accordingly, the first parameter on the list was such allowing the greatest number of malignant lesions to be classified, the second one was such allowing the second greatest number, and so forth. The algorithm then started from the first of the list alone and calculated the corresponding sensitivity, i.e., the percentage of malignant lesions classified as such. The second parameter of the list was then chosen to perform the classification together with the first one, and the sensitivity was computed again. If the second parameter did not allow for the improvement of the classification with at least one more malignant lesion detected, it was discarded as it was considered to be redundant. Otherwise, it was included. Next, the third parameter on the list was added to the first two, and the sensitivity was calculated again, repeating the described process until the addition of more parameters did not improve the sensitivity of malignant lesions.

Thresholds were not set to include all nevi or all melanomas, because preliminary tests showed large values of false positives and false negatives, which would considerably increase the complexity of the algorithm.

After choosing the best statistical descriptors as those allowing more melanomas to be classified as melanomas, a Principal Component Analysis (PCA) [19] was carried out to automatize and enhance the performance of the classification algorithm by avoiding the use of experimental thresholds manually selected.

At first, the matrix of data was standardized. This was done by subtracting the mean and dividing by the standard deviation of every descriptor as follows:

(6) $$Descriptor_{norm}=\;\frac{Descriptor_{Lesion}-mean\left(Descriptor\right)}{stdv\left(Descriptor\right)},$$

Then, Singular Value Decomposition (SVD) [19] was performed over the matrix containing the *d* best descriptors to detect melanomas for every of the 53 lesions (*Y*_dx53_). SVD represents an expansion of the original data in a coordinate system in which the covariance matrix is diagonal. This operation is performed with the *svd* Matlab function obtaining the matrices *U*_dxd_, whose columns correspond to nonzero singular values form a set of orthonormal basis vectors for the range of *Y*; *D*_dx53_, whose diagonal values are the square roots of the eigenvalues; and *V*_53x53_, where the columns are the eigenvectors of the standardized data matrix. *V* therefore contains the principal directions to form a base in which the variance between classes is maximized and *D* the coefficients by which these eigenvectors are multiplied, to obtain the data coordinates transformed into the base of principal directions.

The initial data could be reproduced from the following matrices as follows:

(7) $$\hat{Y}\left(i,j\right)=U\left(i,j\right)\cdot D\left(i,j\right)\cdot V^{t}\left(i,j\right),$$

The first principal components (PCs) can then be calculated as follows:

(8) $$PC_{1}\left(i,j\right)=D\left(1,1\right)\cdot V\left(i,1\right),$$

(9) $$PC_{2}\left(i,j\right)=D\left(2,2\right)\cdot V\left(i,2\right),$$

(10) $$PC_{3}\left(i,j\right)=D\left(3,3\right)\cdot V\left(i,3\right),$$

Many PCs as descriptors originally available are obtained, although the first three PCs are those that are known to contain the higher variability among data (in descendent order).

PCs were represented one against the others, as it is shown in the next section with the aim of transforming the actual descriptors to another coordinate axes where the different classes, nevi, and melanomas are thought to be represented as separate as possible. Additionally, a decision boundary using a Support Vector Machine (SVM) training algorithm was implemented to classify nevi and melanomas [19]. It looks for a hyperplane that separates the space of descriptors into two classes with the maximum margin. This maximum margin is the distance from the decision surface to the closest data point and determines the margin of the classifier. This method of construction necessarily means that the decision function for an SVM is fully specified by a (usually small) subset of the data that defines the position of the separator. These points are referred to as the support vectors; in a vector space, a point can be thought of as a vector between the origin and that point. In the end, the different positions of the separator form the decision boundary from which sensitivity and specificity values can be established. Sensitivity corresponds to the true positive rate defined as the proportion of melanomas that lay inside the boundaries, correctly identified as such; specificity relates to the true negative rate defined as the proportion of nevi that lay outside the boundaries.

## 5. Results and Discussion

**Figure 5:**
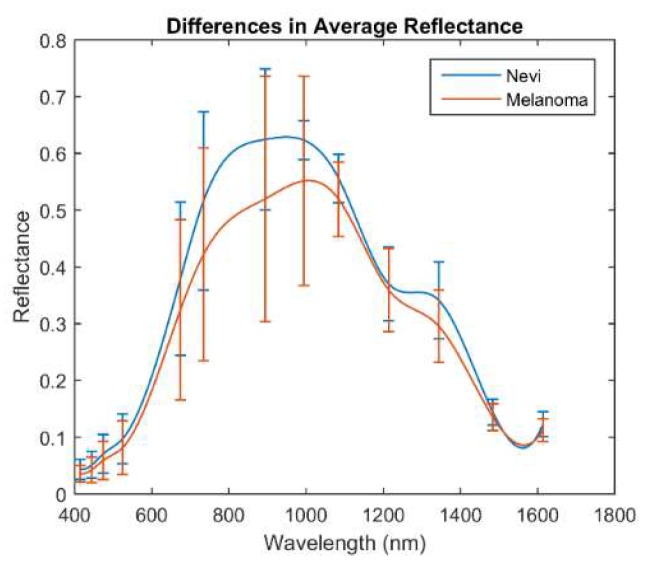
Averaged spectral reflectance curves (±σ) of nevi and melanomas in the VIS-NIR and exNIR ranges.

Figure 5 shows the mean reflectance (±σ) of the whole set of melanomas and nevi measured, separately. The mean reflectance of melanomas in the VIS-NIR and exNIR ranges is lower than that of nevi, being that of nevi, which is especially higher from 995 nm to 1350 nm than for melanomas. This wavelength range, often called second near-infrared window, corresponds to that in which radiation can penetrate deeper into the tissue (longer wavelengths are highly absorbed by water) and thus can inform about deeper structures, which could be notably different between nevi and melanomas.

This behavior agreed with a previous study in which the average reflectance of the nevi was found to be higher with respect to the melanoma population in the VIS range [10], although the standard deviation of the data that was analyzed made it quite entangled.

**Figure 6:**
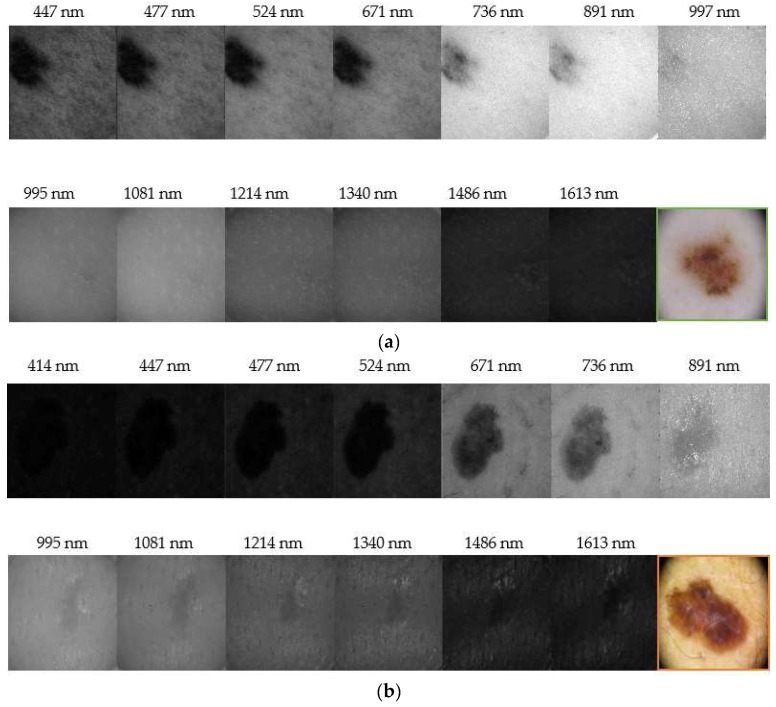
Reflectance images obtained with the systems in the VIS-NIR and exNIR ranges: (**a**) nevus and (**b**) melanoma. (**a**) shows that nevi are usually more homogeneous at all wavelengths. Furthermore, the IR light, which penetrates deeper in the skin, shows that melanomas generally grow deeper in (**b**). Color images were taken with a dermoscope.

Figure 6 depicts representative reflectance images of a nevus and a melanoma for all the spectral range evaluated (414 nm–1613 nm). It can be seen that nevi are usually more homogeneous at all wavelengths, while melanomas grow deeper in the skin as it was found in IR images. Our results correlate with those found by Zhang et al. [20], who identified a strong absorption between 1400 nm and 1450 nm, and also beyond this range, due to the presence of water in tissue, causing a contrast decrease of spectral images. They also evaluated the spectral behavior of tissues for different thicknesses but ex-vivo samples were used; therefore, an accurate comparison with our findings cannot be performed, since we carried out in-vivo measurements.

In the end, the number *d* of best statistical descriptors exhibiting a more accurate classification of lesions that the Matlab algorithm retrieved was eight, corresponding to seven different spectral bands:

- Minimum of the spectral reflectance (minus the average of the healthy skin) at 414 nm.
- Spectral absorbance in terms of the standard deviation at 477 nm.
- Spectral absorbance in terms of the energy at 477 nm.
- Spectral reflectance in terms of energy at 524 nm.
- Spectral reflectance in terms of the skewness at 671 nm.
- Spectral reflectance in terms of the mean at 995 nm.
- Spectral absorbance (minus the average of the healthy skin) in terms of standard deviation at 1214 nm.
- Minimum of the spectral absorbance at 1613 nm.

Table 1 shows these parameters, how the total sensitivity is increased when a new selected parameter is added to the set in the iterative algorithm, and where the experimental thresholds were set in order to obtain the highest possible values of sensitivity.

Combinations of more parameters did not produce better discrimination between nevi and melanomas, taking into account the manual experimental thresholding defined in the scatterplots.

**Figure 7:**
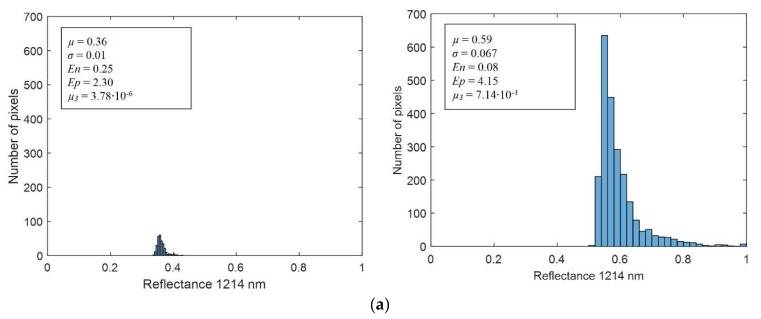

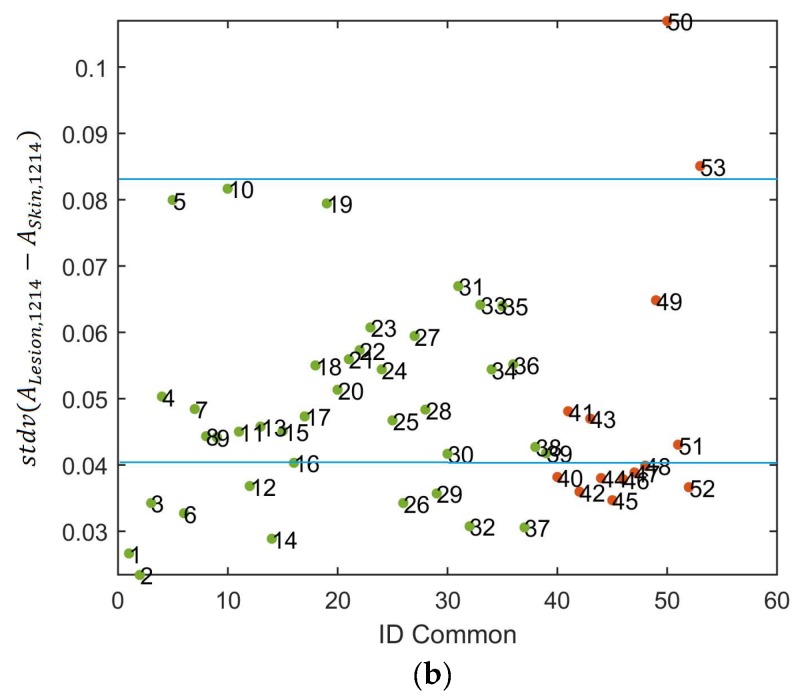
(**a**) Reflectance histogram at 1214 nm including the mean (*µ*), standard deviation (*σ*), energy (*En*), entropy (*Ep*), and skewness or third central moment (*µ*_3_) values of a nevus (left) and a melanoma lesion (right). (**b**) Scatter plot of the spectral absorbance minus the average of the healthy skin in terms of standard deviation at 1214 nm in which manual thresholds have been identified.

Figure 7a shows two representative histograms of a nevus (left) and a melanoma (right) from which the statistical descriptors were computed; they correspond to reflectance images at 1214 nm. The averaged spectral reflectance (*µ*); the standard deviation (*σ*); and corresponding *Ep*, *En,* and *µ*_3_ are also shown. It can be seen that the *En* is lower, and the *Ep* and *µ*_3_ are higher for the melanoma. It means that they are characterized by less uniform images, with a higher disorder in terms of reflectance and a histogram more skewed than that of the nevus. However, it can be appreciated that the *µ* of the melanoma is higher than that of the nevus. Figure 7b depicts a scatter plot of the spectral absorbance minus the average of the healthy skin in terms of standard deviation at 1214 nm. It can be observed that the experimental thresholding was not accurate enough to separate melanomas and nevus.

**Figure 8:**
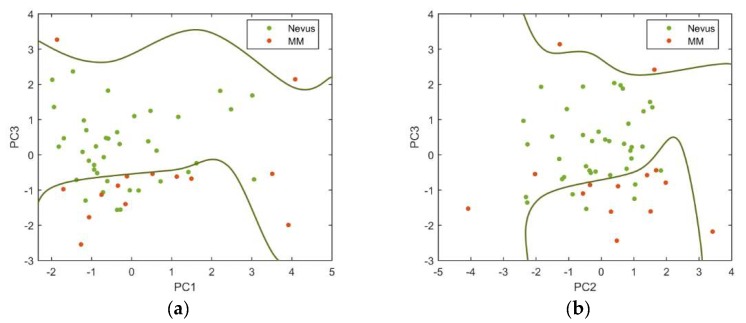
(**a**) PC3 vs. PC1 and (**b**) PC3 vs. PC2 descriptors for nevi (green markers) and melanoma (orange markers) are represented.

In this preliminary study, the first three PCs were found to contain most of the variability among data (98.3%); PC1 explained 73.3%, while PC1 and PC2 explained 83.3% of the variability. Figure 8 shows the PCs plotted against each other to obtain the best descriptor’s space to separate the two types of lesions, as well as the decision boundaries established using a SVM. As it can be seen, the standardization of the descriptors used and the posterior PCA showed to be useful for separating away benign and malignant lesions.

Among all the PCs evaluated, those that produced more precise classifications were the PC3 vs. PC1 (sensitivity = 85.7% and specificity = 76.9%) and the PC3 vs. PC2 (sensitivity = 78.6% and specificity = 84.6%).

Delpueyo et al. found sensitivity and specificity values of 87.2% and 54.5%, respectively, when using only the VIS-NIR device for the analysis of 290 nevi and 95 melanomas [10]. Even though the sensitivity values were similar or slightly lower than when using only the VIS-NIR device [10], the specificity was clearly increased so that exNIR information seems to be relevant for the classification between benign and malignant lesions.

In fact, the detection of malignant lesions at early stages, when they can still be controlled and successfully excised, is crucial when dealing with skin cancer, and this is the reason why dermatologists are more concerned with increasing sensitivity than specificity. However, many of the multispectral systems used for detection of skin cancer in the VIS range generate a large number of false positives and, consequently, a large number of unnecessary biopsies [21,22]. Therefore, the inclusion of longer wavelengths seems to be helpful in order to improve the specificity values reached at the time of the study.

## 6. Conclusions

In this pilot study, a novel exNIR multispectral imaging system for skin cancer diagnosis has been presented. The preliminary analysis of melanomas and nevi from 995 nm to 1613 nm considering spectral and spatial descriptors showed the potential of this technique, particularly for the information obtained from deeper layers of the skin by the use of IR light. In order to improve its performance, the data collected was combined with that provided by a VIS-NIR multispectral imaging system developed in a previous study. The evaluation of lesions from 414 nm to 1613 nm offered an exhaustive spectral assessment of skin lesions.

In addition, the combination of the selected as best parameters for classification, the experimental thresholding, and the use of PCA and SVM enhanced the accuracy of the initial discrimination methodology, leading to similar values of sensitivity but increased ones for specificity. Therefore, exNIR spectral information seems to be relevant for the diagnosis of skin cancer, particularly when nevi and melanomas are taken into account.

The developed methodology was limited by some factors that should be considered for future improvements of the system. Firstly, the resolution of the InGaAs sensor was rather low, limiting the performance of this device. Secondly, the selection of exNIR LEDs was a challenge due to the current state of the art of solid-state technology within this spectral range and the limited availability of wavelengths; their wider FWHMs also contributed to a less accurate spectral reconstruction. Thirdly, the need of the VIS-NIR images for the segmentation of the lesions captured with the exNIR system was also a restriction of this system. Finally, the field of view (FOV) of both systems was another limitation, since only image lesions smaller than 20 mm could be acquired.

The set of lesions was large enough to train the SVM with accurate results but was not to check with other lesions how good the decision boundaries were to separate nevi from melanoma. Future work will be focused on increasing the sample set. Also, combining spectral and 3D data available from a former study [10,23] will increase noticeably the morphological and textural information of lesions and hopefully improve the sensitivity and specificity values. Moreover, usage of the proposed methodology may benefit from conjoint use with the VIS-NIR and exNIR modality to maximize specificity. However, a solution to address the course of action in case of conflict between categorization within the two ranges would be needed. Alternative algorithms considering not only the detection of melanomas but the rate of true positives and true negatives should also be investigated.

## Figures and Tables

**Table 1:** Best statistical descriptors, corresponding experimental upper and lower thresholds, and total cumulative sensitivity.

Parameter	Upper Threshold	Lower Threshold	Cummulative Sensitivity
Min. of the reflectance at 414 nm (minus skin)	0.8	−0.33	36%
Absorbance in terms of *σ* at 477 nm	0.082	0.039	43%
Absorbance in terms of the energy at 477 nm	0.125	0.046	50%
Reflectance in terms of energy at 524 nm	0.28	0.03	64%
Reflectance in terms of the skewness at 721 nm	1.8 × 10^−3^	−2	71.5%
Reflectance in terms of the mean at 995 nm	0.55	0.4	83%
Absorbance (minus skin) in terms of *σ* at 1214 nm	0.082	0.04	91%
Minimum of the absorbance at 1613 nm	1.95	1.18	100%
